# miR-4757-3p Inhibited the Migration and Invasion of Lung Cancer Cell via Targeting Wnt Signaling Pathway

**DOI:** 10.1155/2023/6544042

**Published:** 2023-02-13

**Authors:** Pei Zhao, Qian Zhao, Chen Chen, Song Lu, Li Jin

**Affiliations:** ^1^Department of Intensive Care Unit, Sichuan Cancer Hospital & Institute, Sichuan Cancer Center, School of Medicine, University of Electronic Science and Technology of China, 55# Renmin South Road, Wuhou District, Chengdu 610041, Sichuan, China; ^2^State Key Laboratory of Quality Research in Chinese Medicine and School of Pharmacy, Macau University of Science and Technology, Taipa, China

## Abstract

Lung cancer accounts for the vast majority of cancer-related deaths worldwide, and aberrant miRNA expression is commonly observed as the disease progresses. The current study aimed to determine the role of miR-4757-3p in the development of lung cancer. The real-time PCR test was performed to determine the expression of miR-4757-3p in lung cancer cell lines. miR-4757-3p was downregulated in A549 cells. CCK8 and transwell assays demonstrated that overexpression of miR-4757-3p significantly reduced A549 cell invasion and migration. Bioinformatic analysis by the TargetScan database predicted the possible targets of miR-4757-3p. A luciferase activity test was used to determine the direct relationship between miR-4757-3p, Wnt5a, and Wnt8b. The overexpression of miR-4757-3p drastically inhibited the expression of Wnt5a and Wnt8b. Furthermore, we discovered that silencing Wnt5a and Wnt8b significantly lowered *β*-catenin expression and hampered invasion and migration. Finally, miR-4757-3p inhibited lung cancer cell migration and invasion by inhibiting the activation of the Wnt signaling pathway. Our study provided evidence that miR-4757-3p could be developed as an indicator or an anticancer target in the clinical application.

## 1. Introduction

Over the past few decades, the incidence of lung cancer in both men and women has shown a clear upward trend [1]. In 2020, lung cancer caused 1.8 million deaths worldwide. Lung cancer remains the most common cancer and the leading cause of cancer death in China. The overall 5year survival rate of lung cancer is between 10% and 20% in most countries [2]. The final stage of progression of lung cancer is the unrestrained development and division of abnormal cells [3]. The development of lung cancer is attributable to a variety of factors, including genetic and environmental factors [4]. Lung cancer treatment depends on an in-depth understanding of the cause. The exploration of possible targets to halt the growth and progression of lung cancer aids in the development of lung cancer treatment strategies.

MicroRNAs, also known as miRNAs, are small, noncoding RNAs that are produced in the body and range in length from 21 to 25 nucleotides. miRNAs inhibit protein translation by binding to mismatched sequences in the 3′ untranslated regions of messenger RNAs [5]. After transcription occurs, miRNAs regulate the amount of gene expression produced. Inflammation, viral infection, cancer development, cell division, and apoptosis are all regulated by miRNAs [6]. The abnormal expression of miRNAs in lung cancer may be closely related to the development of cancer and play a role in the pathogenesis of cancer. miRNAs are attractive therapeutic targets, either as oncogenes or as repressors. Previous studies have shown that miR-4757-3p is a target miRNA of Wnt5a or Wnt8b in some tumors [7, 8]. The marker molecules of the Wnt signaling pathway, Wnt5a, and Wnt8b are required for progression of cancer [9]. In this study, we aimed to investigate the function of miR-4757-3p in lung cancer.

## 2. Materials and Methods

### 2.1. Cell Lines and Cell Transfection

The BEAS-2B and A549 cell lines were obtained from the Shanghai Institute of Cell Biology, the China Academy of Sciences (Shanghai, China). Cells were grown in Dulbecco's modified Eagle's medium (DMEM; Sigma, MO, USA) containing 10% fetal bovine serum (FBS) and 1% penicillin-streptomycin solution (Invitrogen, USA). The cells were maintained at 37°C in an incubator. The Lipofectamine 3000 reagent (Invitrogen, USA) was used for transfection with miR-4757-3p mimics (5′-CAUGACGUCACAGAGGCUUCGC-3′), inhibitors (HSTUD1826, Sigma, USA), or miR-NC(5′-CAGUACUUUUGUGUAGUACAA-3′). The media used to cultivate the cells the day before transfection did not include any antibiotics. For siRNA transfection, the cells were transfected with WNT5A-siRNA(5′-GTGGATCAGTTCGTGTGCAAA-3'; Sigma, USA), Wnt8b-siRNA(5′-GCATGGCAGCCTAAACTGCAC-3'; Sigma, USA) or a negative control by using the Lipofectamine 3000 reagent (Invitrogen, USA). After incubation for 48 hours, the cells were collected for further analysis.

### 2.2. Cell Grouping and the CCK-8 Assay

Cell were divided into the following groups: control group (cell transfection with miR-4757-3p mimics/inhibitor NC), miR-4757-3p mimic group (cell transfection with miR-4757-3p mimics), miR-4757-3p inhibitor group (cell transfection with miR-4757-3p inhibitors), siNC group (cell transfection with NC), siWnt5a (cell transfection with Wnt5a-siRNA), siWnt8b (cell transfection with Wnt8b-siRNA), miR-4757-3p inhibitors + siNC (cell cotransfection with miR-4757-3p inhibitors and NC), miR-4757-3p inhibitors + siWnt8b (cell cotransfection with miR-4757-3p inhibitors and Wnt8b-siRNA), and miR-4757-3p inhibitors + Rspo1 (cell transfection with miR-4757-3p inhibitors, while cell incubation with 0.1% Rspo1). The cells were plated into a 96-well plate at a density of 1 × 10^5^ cells per well. The cells were permitted to continue growing for additional 12, 24, or 48 hours. Subsequently, 10 *μ*l of CCK-8 solution was added into cells and incubated for 2 h. The OD_450_ values were determined by using an iMark microplate absorbance reader (BioRad Laboratories, Inc., Hercules, CA, USA).

### 2.3. Transwell Assay

For the migration assay, cells in a serum-free medium were seeded into each well in the upper transwell chamber (Corning USA), and the complete medium was added to the lower chamber. After incubation for 24 h, the cells that were collected using cotton swabs from the upper surface of the membrane migrated to the bottom surface of the membrane. The cells were fixed in 4% paraformaldehyde and stained with 0.1% crystal violet solution. The cells were observed under a microscope (Leica, Germany).

The transwell invasion experiment was performed in the same way as the migration assay, except that 100 mL of Matrigel (BD, USA) diluted 1 : 8 in DMEM was added to each well and incubated at 37°C for 6 hours.

### 2.4. Real-time PCR

Total cellular RNA was extracted by using the TRIzol reagent (Invitrogen, USA). Real-time PCR was performed by utilizing an ABI 7500 fast real-time detection system (Applied Biosystems, Foster City, CA, USA) with SYBR Green I Master Mix (Molecular Probes, Invitrogen) according to the manufacturer's instructions. The relative fold changes were quantified using the delta-delta Ct method with U6 or GAPDH as the endogenous control for normalization. The primers used in real-time PCR are summarized in Table 1.

### 2.5. Western Blot

The proteins in cells were isolated by using the ProteoPrep® total extraction sample kit (Sigma, USA). After centrifuging the cells at a speed of 12,000 g for ten minutes at 4°C, the supernatant was subjected to SDS gel electrophoresis. The proteins were transferred to polyvinylidene fluoride membranes (Millipore, Shanghai, China) and blocked for 1 h at room temperature. The membranes were incubated with antibodies against Wnt5a (#2392, CST, USA), Wnt8b (bs-6245R, Invitrogen, USA), and GAPDH (AG109, Beyotime, China). Subsequently, the membranes were incubated with an anti-mouse IgG HRP-linked antibody (#7076, CST, USA), and the bands were developed by using the ECL detection reagent (Sigma, USA).

### 2.6. Statistical Analysis

Statistical analysis was performed by using SPSS 22.0 software (IBM SPSS Statistics, USA). The data were presented as a mean ± standard deviation (SD). The differences were determined by Student's *t*-test or one-way ANOVA analysis. *P* < 0.05 was considered statistically significant.

## 3. Results

### 3.1. miR-4757-3p Regulated the Migration and Invasion in a549 Cells

**Figure 1:**
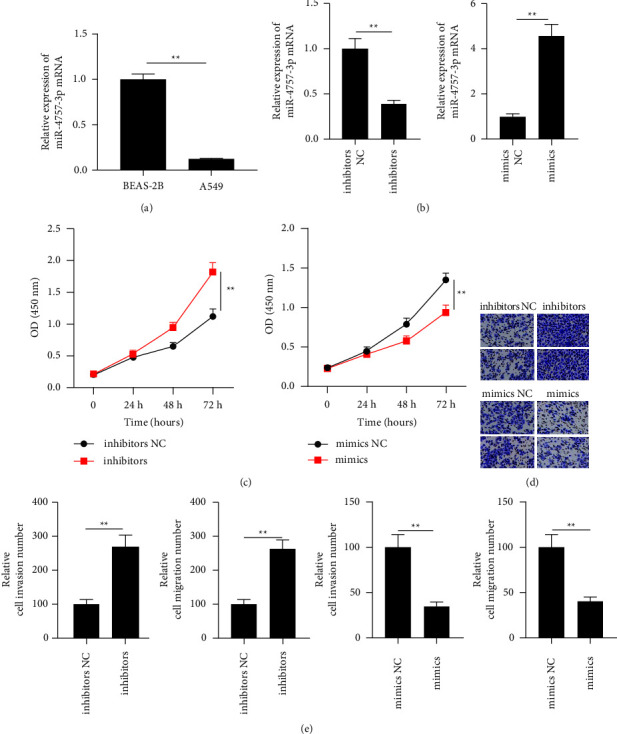
miR-4757-3p regulated the migration and invasion in A549 cells. (a) Real-time PCR analysis of miR-4757-3p expression in A549 cells. (b) Real-time PCR analysis of miR-4757-3p expression in A549 cell transfection with inhibitors or mimics. (c) CCK8 analysis of cell viability in A549 cell transfection with inhibitors or mimics. (d, e) Transwell analysis of cell invasion and migration in A549 cell transfection with inhibitors or mimics. ^*∗∗*^*P*  <  0.01.

**Figure 2:**
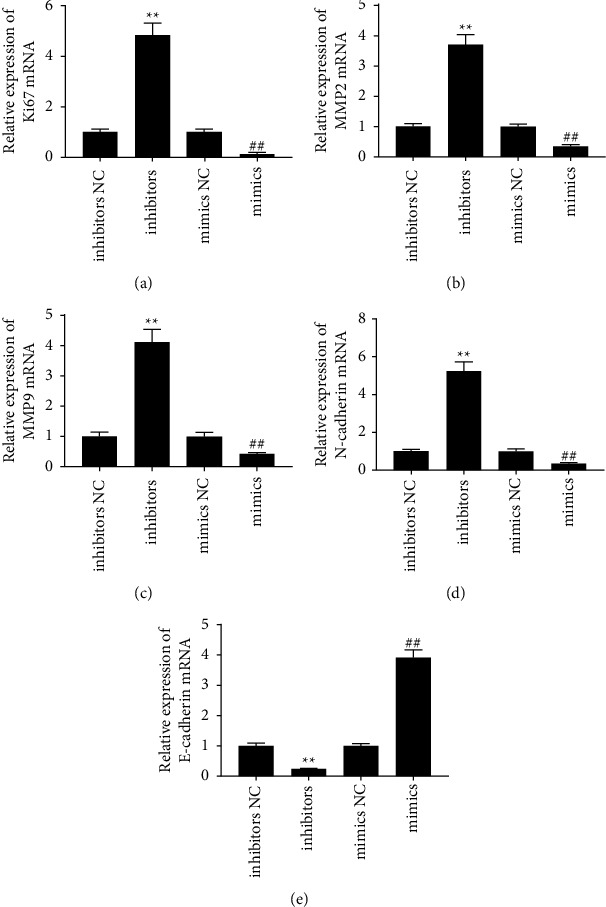
miR-4757-3p regulated gene expression. Real-time PCR analysis of mRNA levels of Ki67 (a), MMP2 (b), MMP9 (c), N-cadherin (d), and E-cadherin (e). ^*∗∗*^*P*  <  0.01 and ^##^*P*  <  0.

The real-time PCR results indicated that miR-4757-3p was downregulated in lung cancer cells (Figure 1(a)). The miR-4757-3p inhibitor and mimic significantly decreased and increased miR-4757-3p levels in A549 cells, respectively (Figure 1(b)). The results of the CCK-8 assay showed that the ability of A549 cells was enhanced after transfection with the miR-4757-3p inhibitor and inhibited after transfection with the miR-4757-3p mimic (Figure 1(c)). Invasion and migration of cells were further determined by the transwell assay. The cell migration and invasion abilities of the miR-4757-3p mimic group were significantly lower than those of the control group, and the cell migration and invasion abilities of the miR-4757-3p inhibitor group were significantly higher than those of the control group (Figure 1(e)). Meanwhile, downregulation of miR-4757-3p increased the expression of Ki67, MMP2, MMP9, and N-cadherin and decreased the expression of E-cadherin (Figures 2(a)–2(e)).

### 3.2. miR-4757-3p Regulated Wnt5a and Wnt8b Expressions and the Activation of the Wnt Signaling Pathway

**Figure 3:**
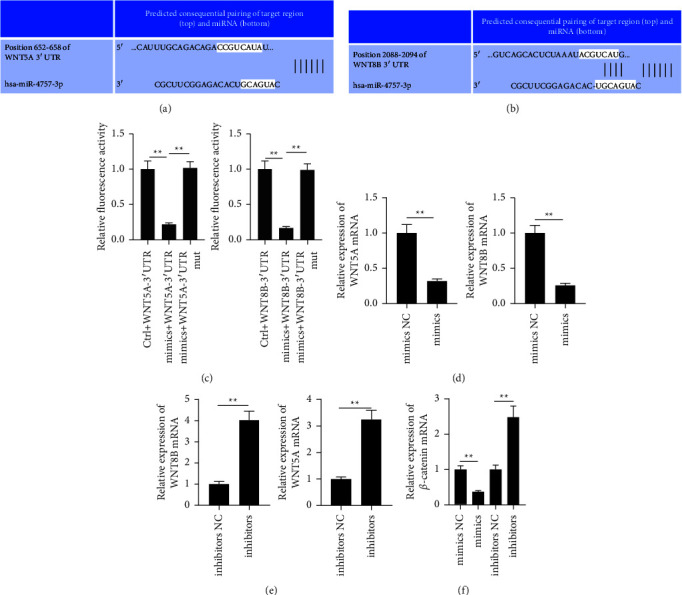
miR-4757-3p regulated Wnt5a and Wnt8b expressions and activated the Wnt signaling pathway. (a) Predicting binding sequences of miR-4757-3p and Wnt5a. (b) Predicting binding sequences of miR-4757-3p and WNT8B. (c) The luciferase reporter assay demonstrated that Wnt5a or Wnt8b 3′ untranslated region-WT was targeted by miR-4757-3p. (d) Real-time PCR analysis of mRNA levels of Wnt5a and Wnt8b in A549 cell transfection with mimics. (e) Real-time PCR analysis of mRNA levels of Wnt5a and Wnt8b in A549 cell transfection with inhibitors. (f) Real-time PCR analysis of *β*-catenin expression in A549 cell transfection with mimics or inhibitors. ^*∗∗*^*P*  <  0.01.

Based on the results of the miRNA target gene prediction database (TargetScan, https://www.targetscan.org/vert_72/), Wnt5a and Wnt8b were identified as target genes of miR-4757-3p (Figures 3(a) and 3(b)). The luciferase reporter assay was employed to validate the above results. The expression of Wnt5a and Wnt8b was significantly decreased in the miR-4757-3p group (Figure 3(c)). Subsequently, the real-time PCR results showed that the expressions of Wnt5a and Wnt8b in the miR-4757-3p mimic group were significantly lower than those in the control group (Figure 3(d)). In contrast, the expression of Wnt5a and Wnt8b in the miR-4757-3p inhibitor group was significantly higher than those in the control group (Figure 3(e)). In addition, compared with the control group, the expression of *β*-catenin was significantly decreased in the miR-4757-3p mimic group, while it was increased in the miR-4757-3p inhibitor group (Figure 3(f)).

### 3.3. Knockdown of Wnt5a or Wnt8b Regulated the Migration and Invasion in A459 Cells and Mediated the Activation of the Wnt Signaling Pathway

**Figure 4:**
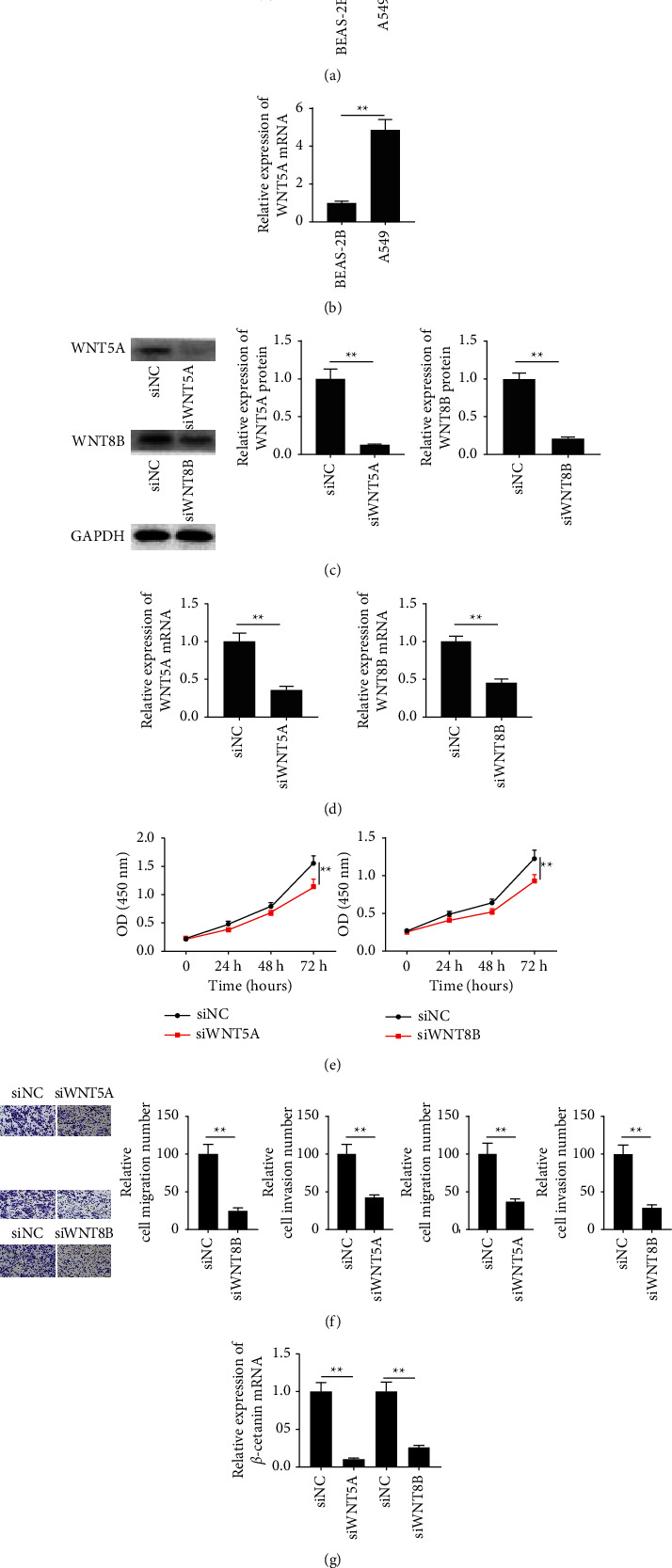
Wnt5a or Wnt8b silencing regulated the migration and invasion in. A459 cells and mediated the activation of the Wnt signaling pathway. (a, b) Real-time PCR analysis of Wnt5a and Wnt8b expressions in A549 cells. (c, d) Western blot and real-time PCR analysis of Wnt5a and Wnt8b expressions in A549 cell transfection with Wnt5a-siRNA or Wnt8b-siRNA. (e) CCK8 analysis of cell viability in A549 cell transfection with Wnt5a-siRNA or Wnt8b-siRNA. (f) Transwell analysis of cell invasion and migration in A549 cell transfection with Wnt5a-siRNA or Wnt8b-siRNA. (g) Real-time PCR analysis of *β*-catenin expression in A549 cell transfection with Wnt5a-siRNA or Wnt8b-siRNA. ^*∗∗*^*P*  <  0.01.

Initially, the expressions of Wnt5a and Wnt8b in the BEAS-2B and A549 cells were compared. As shown in Figures 4(a) and 4(b), both Wnt5a and Wnt8b were highly expressed in A549 cells. The knockdown of two genes by RNA interference on A459 cells was validated by real-time PCR and Western blot. After transfection of Wnt5a-siRNA or Wnt8b-siRNA, the expression of both Wnt5a and Wnt8b in cells was significantly reduced (Figure 4(c)). Furthermore, siWnt5a and siWnt8b significantly reduced cell viability (Figure 4(d)). In addition, knockdown of Wnt5a or Wnt8b was able to inhibit cell migration and invasion (Figure 4(e)) and suppress expression of *β*-catenin (Figure 4(f)).

### 3.4. miR-4757-3p Promoted Cell Migration and Invasion by Targeting Wnt5a and Wnt8b in A549 Cells to Activate the Wnt Signaling Pathway

**Figure 5:**
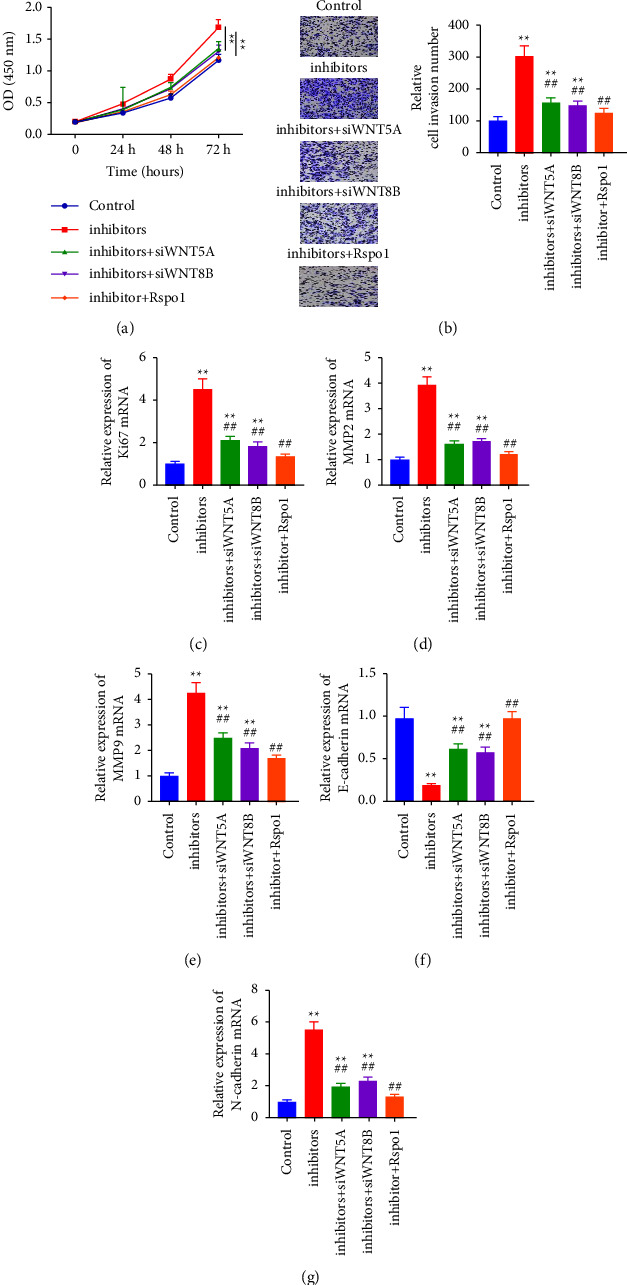
miR-4757-3p promoted cell migration and invasion by targeting Wnt5a and Wnt8b in A549 cells to activate the Wnt signaling pathway. (a) CCK8, (b) transwell analysis of cell viability and invasion, and (c–g) the expression of Ki67, MMP-2/-9, E-cadherin, and N-cadherin in different groups. ^*∗∗*^*P*  <  0.01; ^##^*P*  <  0.01.

As shown in Figure 5, compared with the inhibitor group, cell survival and invasion abilities were improved after cotransfection with the miR-4757-3p inhibitor. The cell viability and invasive capacity of the miR-4757-3p inhibitor group were reduced after cotransfection with Wnt8b-siRNA/or Wnt5a-siRNA (Figures 5(a) and 5(b)). Compared with the miR-4757-3p inhibitor group, cell viability and invasive ability were inhibited. Furthermore, Wnt5a-siRNA, Wnt8b-siRNA, or Rspo1 treatment inhibited the expression of Ki67, MMP-2, MMP-9, and N-cadherin, while they increased E-cadherin expression (Figures 5(c)–5(g)).

## 4. Discussion

Abnormally high proliferation rates, lack of differentiation, and apoptosis are hallmarks of malignancies such as lung cancer, one of the most common types of cancerous tumors [10]. Aberrant expression of miRNAs has the potential to tip the balance in an organism toward the development of cancer [5]. miRNAs can promote the production of oncogenes and reduce the expression of anticancer genes. The expression of target genes has been regulated by miRNA regulatory networks during carcinogenesis and tumor development [11]. No studies indicated that miR-4757-3p was related to cancer, but it was found to be associated with type 1 diabetes. We first examined the expression level of miR-4757-3p, and the results showed that its expression level was relatively low in lung cancer cell lines. Transfection of A549 cells with miR-4757-3p mimics or inhibitors indicated that increasing the levels of miR-4757-3p greatly reduced the ability of cells to migrate and invade. On the other hand, the expression of miR-4757-3p was decreased, resulting in easier migration and invasion of cells. miR-4757-3p may play a role in cell motility and invasion.

To predict the genes targeted by miR-4757-3p, bioinformatic analysis was carried out. The TargetScan database predicted that Wnt5a and Wnt8b genes could bind to miR-4757-3p. The results of the luciferase reporter experiments confirmed their binding. Wnt5a and Wnt8b have been shown to promote cancer progression [12, 13]. Wnt5a and Wnt8b maintain the viability of cancer cells by accelerating the cell cycle, promoting cell proliferation, promoting cell differentiation and senescence, and preventing apoptosis within cells. Wnt5a and Wnt8b are involved in the process of repairing damaged DNA [14]. The Wnt/catenin signaling pathway is often referred to as the canonical Wnt pathway. The Wnt signaling pathway is activated by overexpression of Wnt5a and Wnt8b. It is possible that the Wnt/catenin signaling pathway is persistently activated in an aberrant manner, leading to excessive cell proliferation and transformation into a malignant state [15, 16]. In some cases, this can ultimately result in the development of cancer. A steady low level of catenin is kept in the cells of a healthy organism, and any excess catenin is eliminated by a complex that is made up of proteins, namely, Axin, APC, GSK3, and CK1. Within the degradation complex, the proteins (APC and Axin) serve the function of scaffold proteins, while phosphorylated serine/threonine kinases, GSK3 and CK1, have the ability to phosphorylate catenin [17]. The creation of a complex that includes seven FZD transmembrane protein receptors is the consequence of a connection between factor extracellular Wnt protein and low-density lipoprotein receptor-associated proteins (LRP-5 and LRP-6). It is possible for a Wnt-FZD-LRP5/6 complex to form, and this can lead to the phosphorylation of LRP-6, localization of Axin to the cell membrane, dissociation of GSK3 from Axin and APC, inhibition of catenin degradation, accumulation of catenin, and infiltration of catenin into the cytoplasm and the nucleus of the cell [18–20]. Target genes that are associated with proliferation, invasion, and migration are activated as a result of this continuing connection between nuclear catenin and T lymphocytes/limb-like enhancer and other transcriptional activators [21], leading to abnormally high rates of cell proliferation and metastasis, which, in turn, cause tumors to develop and progress further [22–24]. It was discovered that inhibitors of miR-4757-3p led to a considerable increase in the expression of Wnt5a and Wnt8b. These findings provided evidence that an elevated level of expression of miR-4757-3p can suppress Wnt signaling. In addition, we found that inhibiting Wnt5a and/or Wnt8b activity reduced the amount of cell invasion and migration. A459 cells transfected with miR-4757-3p inhibitors exhibited higher cell migration and invasion in comparison to the control group. However, this effect was reversed when WNT8B expression was reduced by Wnt8b-siRNA/BML-286. Next, the results showed that Wnt8b expression was reduced by Wnt8b-siRNA/Rspo1. Because of targeting Wnt5a and Wnt8b, miR-4757-3p can regulate cell migration and invasion in addition to affecting cell survival.

In conclusion, miR-4757-3p was able to limit the migration and invasion of cells. In addition, the Wnt5a/or Wnt8b interference phenotype was very comparable to the miR-4757-3p phenotype, which was observed in A549 cells. Inhibition of Wnt signaling pathway activation enables miR-4757-3p to regulate Wnt5a and Wnt8b genes to stimulate cell invasion and migration in lung cancer.

## Figures and Tables

**Table 1:** Primer sequences used in this study.

Gene	Forward primer sequence	Reverse primer sequence
miR-4757-3p	AUAGGCCGCUAACGGGC	CCATGACTTGGGGTTACTTAGG
U6	CTGGCCAAAAAGCTTGAAATGGAT	TCGTCCCTAACGCTAGGTCCCT
Ki67	GTGTTAGAGACAAGCGGGGA	TGAAAAGTCGAAGGCGTAGC
MMP2	CAACATACTTTGCATCCTGCAT	AGAGCAGGCAAGCTGGATCTGTG
MMP9	GGCCTTTGGGATCCAGAACGAG	TGTATCCCTGTACACTCTCCAC
E-cadherin	CCACGGCCGACAAATCATCAGCC	GAGCTTCATTGGGTCTCCCTGT
N-cadherin	GTTGCCTATCTCAAATCAAGCCG	TGACGGACTGTCTTGTTTTCACCT
GAPDH	CCCAGGGAGCATTTCGACTGAT	TCACTCGCTCCACAACCCTGT

## Data Availability

Data are available upon request from the corresponding author.
